# Effects of long-term exercise on memory recovery in the aged gerbil hippocampus after transient cerebral ischemia

**DOI:** 10.1186/cc14544

**Published:** 2015-03-16

**Authors:** CW Park, JH Cho, TG Ohk, MC Shin, MH Won

**Affiliations:** 1 Kangwon National Univeristy, Chuncheon-si, Gangwondo, South Korea

## Introduction

Therapeutic exercise is an integral component of rehabilitation for patients with stroke. Despite the high prevalence of cerebral ischemia in the older population, the mechanisms linking restorative exercise to memory recovery from ischemic stroke have not been completely understood in aged animals. In this study, we investigated effects of long-term exercise on neuronal death and memory recovery in the aged gerbil hippocampus after transient cerebral ischemia. We also investigated changes in gliosis, ischemia-induced myelin repair, microvessels, neurogenesis, and growth factor immunoreactivity in the hippocampus to study possible mechanisms of restorative exercise in memory recovery.

## Methods

The gerbils were divided into four groups (*n *= 12 in each group): the sham-operated group (Sham), 4-week sedentary group following ischemia (SD4), 1-week treadmill group following ischemia (TR1) and 4-week treadmill group following ischemia (TR4). Treadmill exercise was stared at 5 days after ischemia/reperfusion (I/R) and lasted for 1 or 4 weeks, and the animals were sacrificed 31 days after ischemia.

## Results

In this study, 4 weeks of treadmill exercise facilitated memory recovery despite neuronal damage in the CA1 region after I/R. On the other hand, the long-term treadmill exercise alleviated the increased gliosis in the CA1 region, and increased the myelin repairing and microvessels in the CA1 region and DG, and enhanced the ischemia-induced cell proliferation, neuroblast differentiation, neuronal maturation of the newly generated cells, and BDNF expression in the ischemic DG of the aged gerbil.

## Conclusion

These results suggest that, in the aged gerbil, long-term treadmill exercise after ischemic stroke could restore the impaired short-term memory function through the cumulative effects of multiple neurorestorative processes.

